# Structure and Dynamics of the Shark Assemblage off Recife, Northeastern Brazil

**DOI:** 10.1371/journal.pone.0102369

**Published:** 2014-07-10

**Authors:** André S. Afonso, Humber A. Andrade, Fábio H. V. Hazin

**Affiliations:** 1 Departamento de Pesca e Aquicultura, Universidade Federal Rural de Pernambuco, Recife, Pernambuco, Brazil; 2 Faculdade de Ciências e Tecnologia, Universidade do Algarve - *Campus* de Gambelas, Faro, Portugal; UC Santa Cruz Department of Ecology and Evolutionary Biology, United States of America

## Abstract

Understanding the ecological factors that regulate elasmobranch abundance in nearshore waters is essential to effectively manage coastal ecosystems and promote conservation. However, little is known about elasmobranch populations in the western South Atlantic Ocean. An 8-year, standardized longline and drumline survey conducted in nearshore waters off Recife, northeastern Brazil, allowed us to describe the shark assemblage and to monitor abundance dynamics using zero-inflated generalized additive models. This region is mostly used by several carcharhinids and one ginglymostomid, but sphyrnids are also present. Blacknose sharks, *Carcharhinus acronotus*, were mostly mature individuals and declined in abundance throughout the survey, contrasting with nurse sharks, *Ginglymostoma cirratum*, which proliferated possibly due to this species being prohibited from all harvest since 2004 in this region. Tiger sharks, *Galeocerdo cuvier*, were mostly juveniles smaller than 200 cm and seem to use nearshore waters off Recife between January and September. No long-term trend in tiger shark abundance was discernible. Spatial distribution was similar in true coastal species (i.e. blacknose and nurse sharks) whereas tiger sharks were most abundant at the middle continental shelf. The sea surface temperature, tidal amplitude, wind direction, water turbidity, and pluviosity were all selected to predict shark abundance off Recife. Interspecific variability in abundance dynamics across spatiotemporal and environmental gradients suggest that the ecological processes regulating shark abundance are generally independent between species, which could add complexity to multi-species fisheries management frameworks. Yet, further research is warranted to ascertain trends at population levels in the South Atlantic Ocean.

## Introduction

Nearshore areas generally comprise shallow, highly productive habitats supporting great abundance and diversity of fish and invertebrates [1], therefore they provide ideal foraging grounds where elasmobranchs can enhance growth [2] and survival [3]–[4]. As a result, several elasmobranchs use coastal waters as nursery grounds [5]–[6], while adults of these species may also exploit these habitats to target high quality prey items which could be unavailable in oceanic waters [7] or to give birth [8]–[9]. Nearshore areas are also used by other species that do not use discrete areas during early life-stages [10] and instead perform wide-ranging movements with little time being spent at any specific location [11], frequently resulting in overlapping distributions of juvenile and mature individuals [10], [12]–[13]. Hence, a combination of life-stages may compose elasmobranch assemblages in nearshore areas, with different species using distinct strategies to enhance population success [14].

On the other hand, nearshore waters typically comprise extremely dynamic ecosystems [15] to which inhabitants must adapt in order to remain in these regions. Highly vagile species such as sharks may cope with environmental variability by accessing coastal waters only when favorable conditions are met and moving away otherwise. Habitat use in coastal sharks has been associated with the tidal cycle [16], water salinity [17]–[18], temperature [19], and storm events [20]. Sharks can thus increase survival by moving away from preferred habitats when facing adverse environmental conditions, and failing to do so could result in mortality [21]–[23]. Moreover, coastal elasmobranchs are also generally exposed to high levels of anthropogenic pressure due to habitat degradation and loss [24]–[27] and fishing. Presumably these anthropogenic impacts will affect elasmobranchs in different ways according to species-specific strategies of habitat use and function.

Sharks are a key-component of coastal ecosystems because they generally act as high-level predators and consume a large portion of available energy [28]. Thus, the depletion of their populations may have striking consequences, such as mesopredator releases and trophic cascades [29]–[30] which may potentially change the structural properties of the ecosystem [31]–[32]. Understanding how species and communities use nearshore areas is of utmost importance so that effective conservation and management can be implemented. On that account, assessing the spatiotemporal variability in community structure is a first step to elucidate ecological processes in elasmobranchs [33]. The strategy a species utilizes to maximize survival is shaped by both its life-history characteristics [34]–[35] and by a combination of ecological factors including environmental features, resource abundance and distribution, and the presence of predators and/or competing species [36]–[39]. This frequently results in high interspecific variability in distribution [14] and behavior [40]. Identifying the factors that regulate the dynamics of the elasmobranch community should thus improve the efficiency of conservation measures, particularly in previously unstudied regions such as the western South Atlantic Ocean.

This study aims at characterizing the shark assemblage off the Metropolitan Region of Recife and assessing its spatiotemporal dynamics together with the environmental factors that regulate species abundance in order to understand species-specific trends in the use of nearshore areas. The results obtained allowed us to describe the population structure of the most abundant species and to identify the factors that interact with the abundance of each species in these coastal habitats.

## Materials and Methods

### Ethics statement

The data used in this research was obtained with full approval of the Instituto Chico Mendes de Conservação da Biodiversidade of the Brazilian Ministry of the Environment (permit no. 15083–8), which included authorization to sample a protected species, i.e. the nurse shark *Ginglymostoma cirratum*.

### Sampling procedure

**Figure 1:**
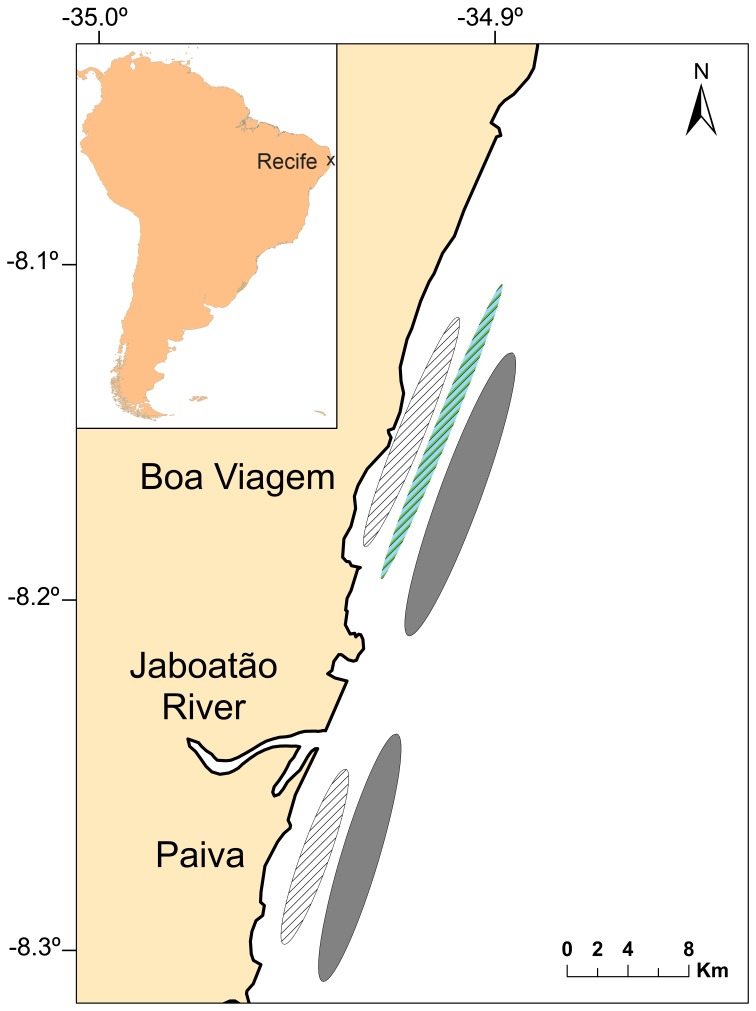
Study area. Map of the littoral of Recife, northeastern Brazil, depicting the locations of a shallow alongshore reef (stripped blue ellipse off Boa Viagem) and both bottom longline (solid gray ellipses located seaward) and drumline (blank striped ellipses located shoreward) deployments in two nearshore fishing sites.

This study used data from a longline and drumline survey targeting large sharks off Recife (8°10′S, 34°53′W), northeastern Brazil, from May 2004 to December 2011 [41]–[42]. The study area comprised two adjacent, nearshore fishing sites, hereafter referred to as Boa Viagem (BV) and Paiva (PA), between the 3-m and 18-m isobaths (Fig. 1). BV is a widely urbanized beach and has greater habitat complexity due to the presence of an alongshore, shallow reef [42], whereas PA is a comparatively undeveloped region with a relatively monotonous bathymetric profile that includes the Jaboatão estuary in its northernmost section. A total of 1,130 fishing cruises, generally comprising four consecutive fishing sets in each site, were conducted on a weekly basis. Bottom longline gear was deployed late afternoon and retrieved in the following dawn, whereas drumlines were inspected at dawn for bait refurbishment. Longlines were composed of a 4-km long mainline with 100 hooks and were deployed alongshore, ∼1.5−3 km away from the coastline (Fig. 1). Drumlines, numbering 13 off BV and 10 off PA, were composed of an 18-m long, vertically-stretched mainline with 2 hooks and were deployed ∼0.5−1 km from the coastline. Additionally, 38 bottom longline sets (200 hooks each) were occasionally conducted at the middle continental shelf (CS) between the 25- and 40-m isobaths. Altogether, the fishing effort in this study totaled 280,079 deployed hooks. Circle hooks (17/0, 10° offset) baited mostly with *Gymnothorax* moray eel (∼300 g) were used, but J-style hooks (9/0, 0° offset) were also used until May 2006 for hook-performance comparison [43]. Also, a Styrofoam float was attached to the proximal end of terminal tackles in order to suspend all hooks in the water column since September 2005. Yet, because such modification significantly influenced catchability [43], the period from May 2004 to August 2005 was discarded from abundance analyses. All fishing sets followed the same rigorous methodology so that the influence of fishing gear and procedure on species catchability could be standardized. Further details on the fishing methodology and fishing effort are thoroughly described in [42], whereas an environmental description of the study area can be found in [41]. While both longlines and drumlines were used for fishing, drumline data were discarded from abundance analyses because both fishing gears had distinct efforts and spatial arrangements which could potentially confound interpretation of catch rate data.

All sharks caught were identified, sexed, and measured for stretched total length (TL) to the nearest centimeter. Several environmental parameters were monitored after deploying and retrieving the longline gear. Sea surface temperature (±0.01°C) and salinity (±0.1 ups) were measured with a YSI 556 multiprobe. Water transparency (±0.5 m) was measured with a Secchi disc. Tidal amplitude (±0.1 m) for the Port of Recife was obtained from the Hydrographic and Navigation Directory of the Brazilian Navy (http://www.mar.mil.br/dhn/chm/tabuas/index.htm). The day of the lunar cycle was obtained from http://kalender-365.de/calendario-lunar-pt.php, with the new-moon day corresponding to the first day of the cycle. Meteorological variables such as daily pluviosity (mm), wind direction (0−360°) and speed (m·s^−1^), and cumulative solar radiation (kW·h·m^−2^) were obtained from the Center for Weather Forecasting and Climate Studies of the National Institute for Space Research (http://sinda.crn2.inpe.br/PCD/historico/consulta_pcdm.jsp) for the region of Recife between May 2004 and December 2011.

### Statistical analyses

Statistical analyses were performed on the most abundant shark species (>50 individuals caught). Size and sex compositions were assessed for each of such species and differences in mean total length between males and females were assessed with 2-sample t-tests. Deviances from the 1∶1 sex ratio were assessed with chi-square goodness-of-fit tests. Kruskal-Wallis rank sum tests were used to compare total lengths between years and between quarters. Whenever significant differences between quarters were detected, a post-hoc, multiple comparison procedure [44] was used to investigate which quarters were different. Pearson’s chi-square tests were used to detect significant shifts in sex ratio across years and quarters for each species.

Because longline sets within fishing cruises could not be considered independent sampling [42], catch and effort data were aggregated by fishing cruise and environmental variables were averaged by fishing cruise for abundance analyses. A 2-sample t test was used to assess for differences in longline soak time between nearshore fishing sites. A total of 12 candidate predictors of species abundance were considered: *year*, *month*, *fishing site*, *lunar day*, *sea surface temperature*, *salinity*, *visibility*, *tidal amplitude*, *pluviosity*, *wind direction*, *wind speed*, and *cumulative solar radiation*. All predictors but *fishing site* were interpreted as continuous variables. Further details on predictor variables, including the abbreviations hereafter used, can be found in Table 1. Possible correlations between predictors were investigated in order to avoid including correlated variables in the same model. Spearman’s rank correlation coefficient, *s*, was assessed for all pairwise combinations of continuous predictors. Additionally, the significance of Pearson’s product-moment correlation coefficient, *r*, was assessed using Student’s t distribution with *n* − 2 degrees of freedom to test the null hypothesis *ρ* = 0 [45]. Also, 95% confidence intervals for *r* were calculated using Fisher’s *Z* transformation [46]. Both procedures were conducted using the *cor.test* function in STATS *R*-library [47]. Correlation coefficients lower than 0.3 were considered small [48]. The existence of correlation between predictors were identified when three criteria were met, namely *i*) the null hypothesis that *ρ* = 0 was rejected (*p*<0.05), *ii*) the highest absolute value in the confidence interval for *ρ* was greater or equal than 0.3, and *iii*) either the absolute value of *s* or the lowest absolute value of the confidence interval for *ρ* were greater or equal than 0.3. Whenever a problematic correlation was detected, the responsible covariates were not used simultaneously in any model. Although the value 0.3 is subjective in the sense that any other low value could be used, it proved to be effective because it allowed us to discard the most correlated covariates while preserving nearly 80% of the combinations between weakly correlated or uncorrelated predictors.

**Table 1:** Selected predictive variables.

Variable	Abbreviation	Type	Description
Site	*site*	Categorical	Boa Viagem (BV), Paiva (PA)
Year	*year*	Continuous	2005−2011
Month	*month*	Continuous	1−12
Lunar day	*lunday*	Continuous	The day number of the lunar cycle, starting in new-moon day
Temperature	*temp*	Continuous	Sea surface temperature, in degrees
Salinity	*salin*	Continuous	Practical salinity units
Visibility	*visib*	Continuous	Water visibility, in meters
Tidal amplitude	*tidamp*	Continuous	Difference between highest and lowest tidal height per day
Pluviosity	*pluvio*	Continuous	Rainfall in milimeters
Wind direction	*winddir*	Continuous	Direction in 0−360 degrees, clockwise
Wind speed	*windspe*	Continuous	Velocity in meters per second
Cumulative solar radiation	*solarrad*	Continuous	Total solar radiation per day, in kiloWatts·hour per square meter

Description of the predictive variables used to model elasmobranch abundance off Recife.

Modeling the abundance of sharks is often complicated by a large amount of zero-valued observations, which may yield zero-inflated distributions [49]. A general approach to nonparametric regression analysis with zero-inflated data consists on modeling the response distribution as a probabilistic mixture of zero and a regular component whose distribution belongs to the exponential family [50]. Generalized additive models (GAM) are widely used for modeling nonlinear effects of covariates in quantitative studies [51]–[52] and can be extended for such data, resulting in zero-inflated generalized additive models (ZIGAM) [53]–[54]. However, the ZIGAM approach implicitly assumes that the zero-inflation process is uncoupled from the regular model component, which may not always be true. A recently developed alternative, the constrained zero-inflated generalized additive model (COZIGAM) approach, implicitly assumes that the probability of non-zero inflation and the mean non-zero-inflated population abundance are linearly related on some link scales [55].

Catch data for each species were fitted against each of the predictive variables individually using GAM and ZIGAM to assess if the distribution of the data was zero-inflated. While zero-inflated models proved to be the best alternative, the COZIGAM was also fitted to the data in order to make comparisons with the larger (more parameters) ZIGAM. This allowed us to verify the independence of the non-zero-inflated data generation process relative to the zero-inflated process. The type of model which generally exhibited best performance was selected for the analysis. Modeling was conducted with COZIGAM *R*-library [55]. The Poisson distribution was used to model the non-zero-inflated process, whereas the binomial distribution was used to model the zero-inflated process. The thin-plate regression spline was used as a penalized smoothing basis, and the *k* dimensions of the basis representing the smoothing terms were optimized for each predictor variable by running several univariate models with different *k* values and comparing their output. Parameter estimates were obtained with the EM algorithm [56] because typical procedures to obtain parameter estimates cannot be used when the state (i.e., the zero-inflated or the non-zero-inflated processes) which the zero-valued observations belong to is unknown [57]. A maximum of 250 interactions were allowed to occur for the algorithm to converge. The logarithm of fishing effort was included in the model as an offset covariate for standardization of the catch rate.

Given the particular nature of the covariate *month*, which may yield significant correlations with environmental variables most notably when seasonality is present, modeling was approached in two separate forms: the spatiotemporal model (*SPT*), which includes the covariates *year*, *month*, and *site*; and the environmental model (*ENV*), which includes the remaining covariates that are not correlated. Regarding *SPT* modeling, two different approaches were conducted, more precisely *i) SPT1*, comprising *site* as a factorial covariate and covariates *year* and *month* as independent smooth functions, and *ii) SPT2*, comprising *site* as a factorial covariate and covariates *year* and *month* linked by the same smoothing spline. Regarding *ENV* modeling, *site* was also included as a factor because the catch rates of some species were found to be significantly different between fishing sites. Predictive variables with higher effect on abundance were selected to be included in the *ENV* model with a forward stepwise approach [58]. The Bayesian approximated logarithmic marginal likelihood by Laplace method, *logE*, was used for model comparisons and selection [55]. All statistical analyses were conducted in *R* version 2.14.0 [47].

## Results

The shark assemblage surveyed by the present study comprised seven carcharhinids, two sphyrnids, and one ginglymostomid (Table 2). The catch composition was clearly dominated by three species, i.e. the nurse, *Ginglymostoma cirratum*, the blacknose, *Carcharhinus acronotus*, and the tiger, *Galeocerdo cuvier*, sharks, with 149, 125 and 56 individuals caught, respectively. The bull, *Carcharhinus leucas*, and the blacktip, *C*. *limbatus*, sharks were infrequently caught, whereas the silky, *C*. *falciformis*, the Caribbean reef, *C. perezi*, the Brazilian sharpnose, *Rhizoprionodon lalandii*, and both the scalloped and great hammerheads, *Sphyrna lewini* and *S*. *mokarran*, were rarely caught.

**Table 2:** Summary of shark species.

Species	*N_t_*	Total length (cm)				Sex ratio
		Min	Max	Mean	S.D.	M:F (*N_s_)*
*Ginglymostoma cirratum*	149	92*	300*	189.0*	43.5*	0.78∶1 (116)*
*Carcharhinus acronotus*	125	39	180	111.8	16.1	0.77∶1 (122)
*Galeocerdo cuvier*	56	82	355	158.2	58.4	0.69∶1 (56)
*Carcharhinus leucas*	11	144	250	193.7	32.5	0.67∶1 (11)
*Carcharhinus limbatus*	6	80	209	125.7	53.3	1∶1 (6)
*Carcharhinus falciformis*	2	83	126	104.5	30.4	1∶1 (2)
*Carcharhinus perezi*	1	107	107	−	−	0∶1 (1)
*Rhizoprionodon lalandii*	1	51	51	−	−	0∶1 (1)
*Sphyrna mokarran*	1	346	346	−	−	1∶0 (1)
*Sphyrna lewini*	1	222	222	−	−	1∶0 (1)

Total lengths (minimum, maximum, mean, and standard deviation) and sex ratio, as the ratio between males and females, of sharks caught off Recife, Brazil between 2004 and 2011. *N_t_* and *N_s_* denote the number of individuals caught and sexed, respectively.

*Only includes sharks caught since October 2007.

### Size composition

**Figure 2:**
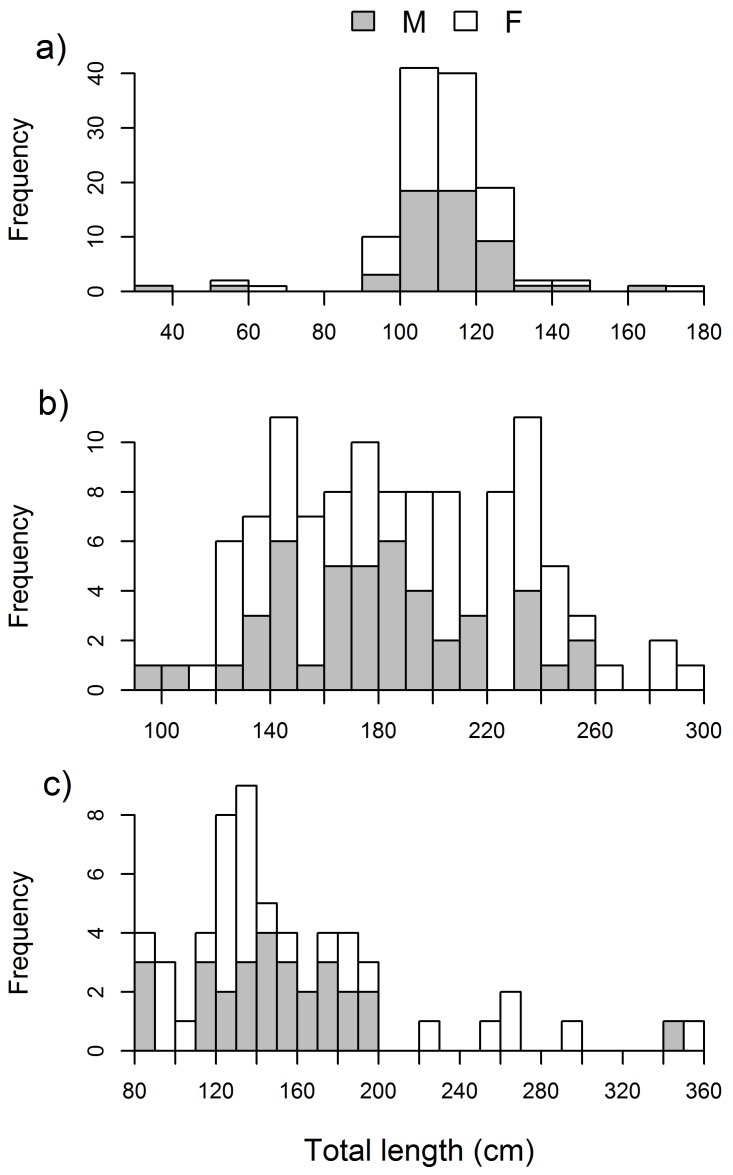
Size-structure of abundant sharks. Absolute frequencies of 10-cm total length-classes, divided in male (gray) and female (blank) components, for a) blacknose sharks, b) nurse sharks, and c) tiger sharks caught off Recife, Brazil, between 2004 and 2011.

Among the most abundant *taxa*, nurse sharks had the largest mean TL and blacknose sharks the smallest, but tiger sharks attained the largest size and size range (Table 2). Tiger sharks also attained the largest size among carcharhinids but bull sharks had the largest mean TL. The remaining carcharhinids were generally small but sphyrnids measured >200 cm TL. Regarding length-frequency distributions, blacknose sharks exhibited a distinct mode, with 68% of the individuals measuring 100−120 cm TL and 92% measuring 90−130 cm TL (Fig. 2a). Nurse sharks measuring 120−240 cm TL were uniformly abundant and totaled 91% of the nurse shark catch, but they ranged between 92 and 300 cm TL with females prevailing at sizes ≥220 cm TL (Fig. 2b). Juvenile tiger sharks of both sexes measuring 82−200 cm TL comprised 88% of the tiger shark catch, whereas sharks ≥220 cm TL were mostly females (Fig. 2c). Similarly, the largest bull and blacktip sharks were females. No significant differences in mean TL between sexes were found for blacknose (t = −0.093, *p* = 0.926), nurse (t = −1.366, *p* = 0.175), or tiger (t = −0.453, *p* = 0.653) sharks, thus both sexes were pooled together for length analyses.

**Figure 3:**
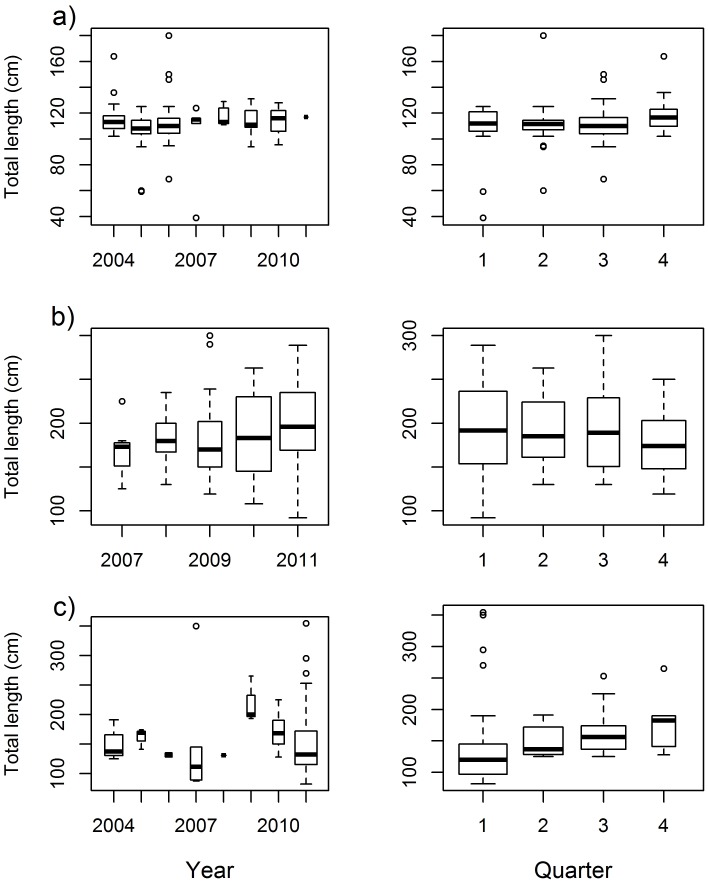
Temporal variability in shark size. Distribution of total lengths per quarter and per year for a) blacknose shark, b) nurse shark, and c) tiger shark. In each plot, box width is proportional to the square root of the number of individuals measured.

**Figure 4:**
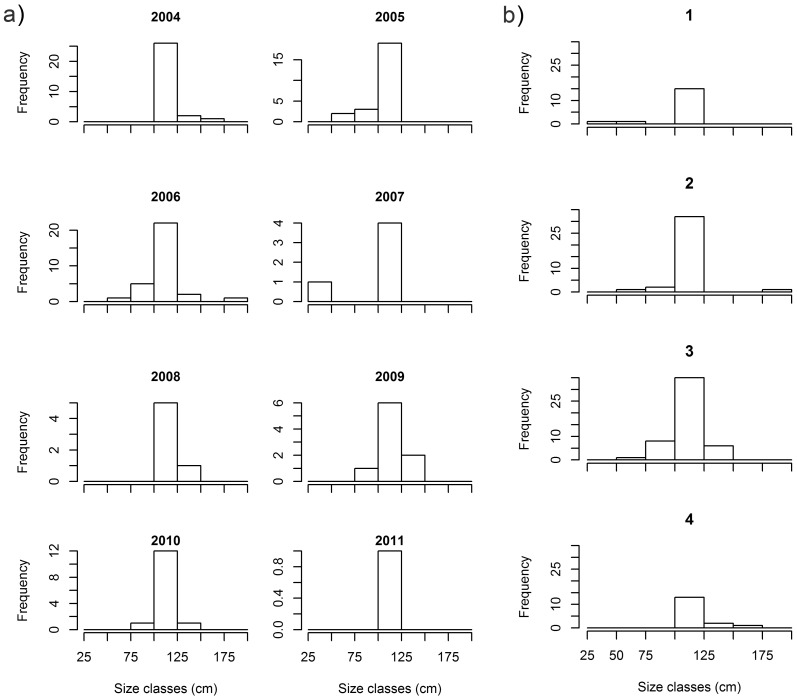
Dynamics in blacknose shark length-frequency distribution. Absolute frequencies of blacknose shark total lengths in 25-cm size classes across a) years, and b) quarters.

**Figure 5:**
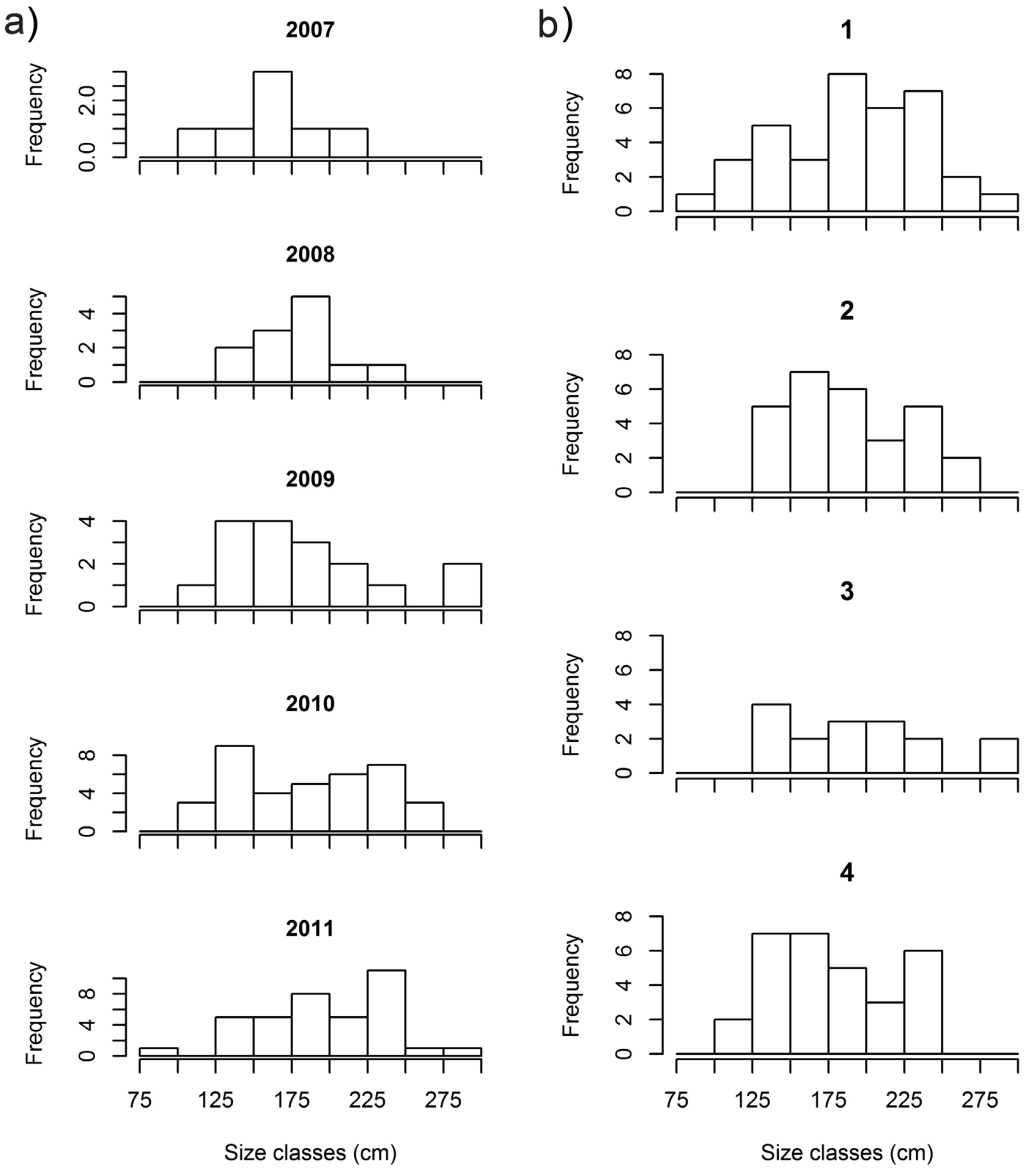
Dynamics in nurse shark length-frequency distribution. Absolute frequencies of nurse shark total lengths in 25-cm size classes across a) years, and b) quarters.

**Figure 6:**
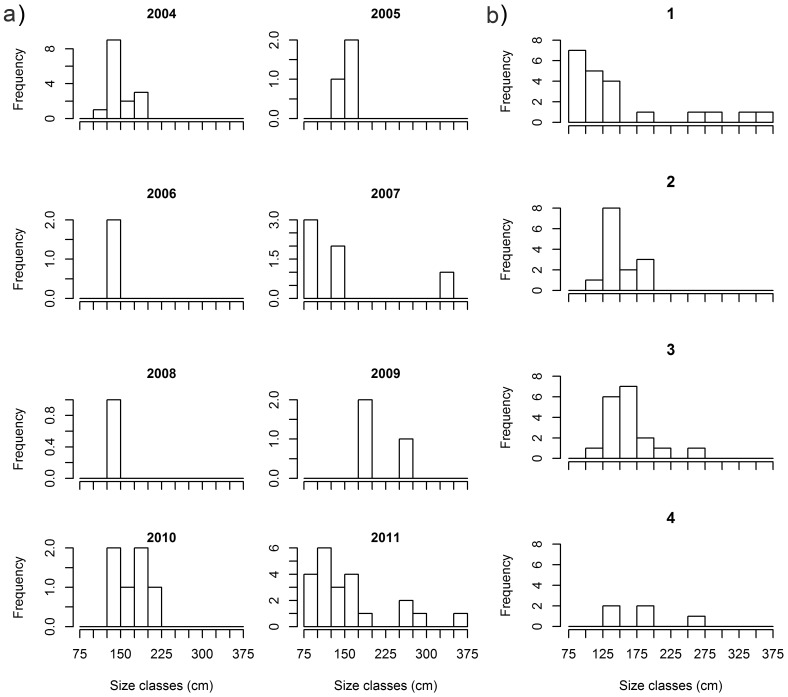
Dynamics in tiger shark length-frequency distribution. Absolute frequencies of tiger shark total lengths in 25-cm size classes across a) years, and b) quarters.

Blacknose sharks showed little variation in size across years and quarters (Fig. 3a; Fig. 4a). Smaller sharks occurred between the first and third quarters and larger sharks occurred mostly between the third and fourth quarters (Fig. 4b), but no differences between quarters (χ^2^ = 4.601; *p* = 0.204) or years (χ^2^ = 8.103; *p* = 0.324) were detected. Nurse shark median size and range increased from 2007 through 2011 (Fig. 3b), with sharks <100 cm TL occurring in 2011 only (Fig. 5a) and the first quarter showing highest variability in shark size (Fig. 3b; Fig. 5b), but no significant differences between quarters (χ^2^ = 1.527; *p* = 0.676) or years (χ^2^ = 5.188; *p* = 0.269) were found. Tiger shark abundance showed annual fluctuations that resulted in small sample sizes in most years, precluding the assessment of annual trends in shark size (Fig. 3c). Yet, sharks ≥250 cm TL were caught in 2007, 2009 and 2011 only (Fig. 6a). On the other hand, tiger shark size increased throughout the year (Fig. 3c), as indicated by a modal progression in length-frequency distribution from the first through the third quarters (Fig. 6b). Tiger sharks <100 cm TL occurred exclusively in the first quarter, when 76% of the sharks measured less than 150 cm TL. However, the largest individuals also occurred during this period. The mode then shifted to the 125−149 and 150−174 cm TL size-classes in the second and third quarters, respectively, whereas only a few medium-sized juveniles were caught in the fourth quarter. A Kruskal-Wallis test detected significant differences in tiger shark size between quarters (χ^2^ = 9.131; *p* = 0.028), and a post-hoc procedure indicated the first and the third quarters to be different (Diff._Obs_ = 14.877; Diff._Cri_ = 14.310).

### Sex ratio

**Figure 7:**
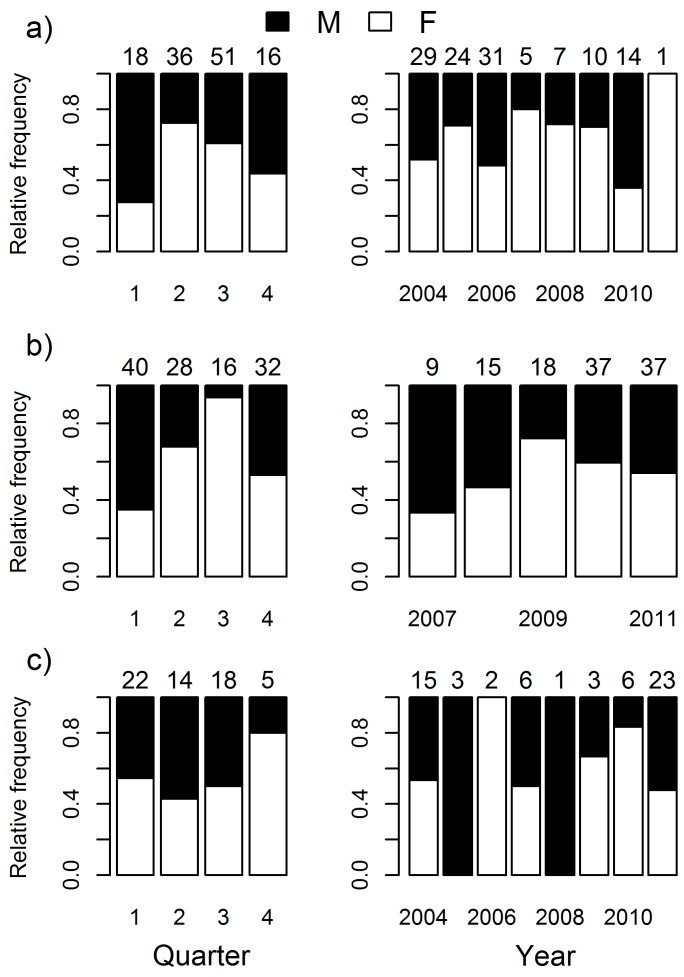
Sex proportion dynamics. Variation of the relative frequency of male (solid bars) and female (blank bars) a) blacknose sharks, b) nurse sharks, and c) tiger sharks, between quarters (left panels) and years (right panels). Numbers above bars correspond to the number of sharks caught in the respective period. Note that nurse sharks were not sexed before 2007.

The male:female ratio of blacknose sharks equaled 0.77∶1 (Table 2) and did not deviate significantly from 1∶1 (χ^2^ = 2.098, df = 1, *p* = 0.148). However, males were relatively more frequent in the first quarter, when catch was low, whereas females were relatively more frequent in the second and third quarters when catch was high (Fig. 7a). Significant differences were detected between quarters (χ^2^ = 11.120, df = 3, *p* = 0.011) but not between years (χ^2^ = 8.848, df = 7, *p* = 0.264). The nurse shark sex ratio was 0.78∶1 (Table 2) and did not deviate from 1∶1 (χ^2^ = 1.6897, df = 1, *p* = 0.1936). Males predominated in the first quarter, when catch was high, but females prevailed in the second quarter and, more strikingly, in the third quarter when catch was particularly low (Fig. 7b). Significant differences were detected between quarters (χ^2^ = 18.121, df = 3, *p*<0.001) but not between years (χ^2^ = 4.567, df = 4, *p* = 0.335). Tiger shark sex ratio equaled 0.69∶1 and did not deviate from 1∶1 (χ^2^ = 0.153, df = 1, *p* = 0.696). No trend was discernible in sex ratio variation (Fig. 7c) and statistical tests detected no effect for years (χ^2^ = 8.981, df = 7, *p* = 0.254) or quarters (χ^2^ = 2.121, df = 3, *p* = 0.548).

### Patterns and dynamics in abundance

After aggregating fishing sets by fishing cruise, a total of 518 samples equally distributed between the two nearshore sites, BV and PA, plus 38 samples from the middle continental shelf (CS) were considered for abundance analysis. Positive catch equaled 16% for nurse sharks, 9% for blacknose sharks and 6% for tiger sharks. Univariate models for all variables and for each species revealed that ZIGAM always had higher *logE*’s than GAM (Table 3), thus confirming zero-inflation in data distribution. Further univariate comparisons between ZIGAM and COZIGAM revealed that ZIGAM exhibited higher *logE*’s for virtually all variables (Table 3), thus the non-constrained version of the zero-inflated model was chosen to model species abundance off Recife.

**Table 3:** Model-type comparisons.

Species	Predictor	GAM	ZIGAM	COZIGAM
*Carcharhinus acronotus*	Year	−203.526	−**186.97**	−371.308
	Month	−233.725	−**192.835**	−305.444
	Lunar day	−231.402	−**206.348**	−213.932
	Temperature	−231.379	−**192.142**	−297.501
	Salinity	−206.893	−**193.537**	−214.203
	Visibility	−231.569	−**203.118**	−214.324
	Pluviosity	−231.491	−**207.035**	−217.949
	Tidal amplitude	−231.438	−206.501	−**204.998**
	Wind direction	−223.608	−**197.384**	−200.750
	Solar radiation	−230.833	−**206.058**	−218.846
	Wind speed	−211.495	−**193.497**	NA
*Ginglymostoma cirratum*	Year	−297.338	−**282.258**	−815.652
	Month	−310.557	−**300.78**	−540.733
	Lunar day	−312.917	−**302.46**	−435.343
	Temperature	−309.956	−**302.012**	−545.362
	Salinity	−306.540	−**290.829**	−296.893
	Visibility	−299.013	−**288.377**	NA
	Pluviosity	−312.857	−**303.905**	NA
	Tidal amplitude	−312.821	−**303.751**	NA
	Wind direction	−309.104	−**296.706**	−829.089
	Solar radiation	−313.923	−**294.462**	−797.744
	Wind speed	−309.396	−**290.800**	−302.599
*Galeocerdo cuvier*	Year	−127.749	−**124.151**	−260.078
	Month	−132.918	−**126.782**	−171.675
	Lunar day	−133.486	−**130.147**	−346.171
	Temperature	−136.727	−**132.579**	NA
	Salinity	−133.734	−**131.848**	−368.931
	Visibility	−131.901	−**126.988**	−471.958
	Pluviosity	−135.513	−**125.94**	NA
	Tidal amplitude	−135.688	−**119.908**	NA
	Wind direction	−129.589	−**117.531**	−462.808
	Solar radiation	−135.297	−**132.786**	−279.408
	Wind speed	−124.665	−**120.939**	−274.774

Approximated logarithimic marginal likelihoods, *logE*, of single models with one predictor variable for each species, assessed with non-inflated Generalized Additive Models (GAM), zero-inflated Generalized Additive Models (ZIGAM), and constrained zero-inflated Generalized Additive Models (COZIGAM). The lowest *logE* for each species and predictor is typed in bold face. NA’s correspond to unsuccessfully fitted models which did not converge.

Correlation analyses between environmental variables detected problematic correlations between *visib* and *temp*, *windspe*, and *winddir*, and between *pluvio* and *winddir* (Table 4), thus these variables were not included simultaneously in the same model. Although longline soak time was significantly different between fishing sites (*t* = 8.543, df = 1134, *p*<0.001), the average magnitude of such difference (∼1 h) was small (∼7%) compared to average soak time (14−15 h) (Hazin & Afonso 2013).

**Table 4:** Summary of correlation analyses to assess variable interdependencies.

Covariate 1	Covariate 2	t-statist.	d.f.	*p*-value	LL	UL	*r*	*s*
Temperature	Salinity	2.768	241	0.006	0.051	0.295	0.176	0.358
**Temperature**	**Visibility**	**13.333**	**332**	**<0.001**	**0.516**	**0.656**	**0.591**	**0.600**
Temperature	Tidal amplitude	0.230	452	0.818	−0.081	0.103	0.011	0.016
Temperature	Pluviosity	−4.104	408	<0.001	−0.290	−0.104	−0.199	−0.203
Temperature	Wind speed	−0.432	418	0.666	−0.117	0.075	−0.022	−0.034
Temperature	Wind direction	−4.267	418	<0.001	−0.294	−0.111	−0.204	−0.191
Temperature	Solar radiation	4.038	349	<0.001	0.109	0.309	0.211	0.242
Salinity	Visibility	0.162	223	0.872	−0.120	0.141	0.011	0.198
Salinity	Tidal amplitude	0.836	241	0.403	−0.073	0.178	0.054	0.036
Salinity	Pluviosity	−2.991	226	0.003	−0.317	−0.067	−0.195	−0.211
Salinity	Wind speed	2.569	228	0.011	0.039	0.291	0.168	0.206
Salinity	Wind direction	−0.404	228	0.687	−0.156	0.103	−0.027	−0.078
Salinity	Solar radiation	2.812	202	0.005	0.058	0.323	0.194	0.151
Visibility	Tidal amplitude	−0.131	376	0.896	−0.108	0.094	−0.007	0.009
Visibility	Pluviosity	−3.404	336	<0.001	−0.284	−0.077	−0.183	−0.172
**Visibility**	**Wind speed**	−**11.54**	**342**	**<0.001**	−**0.601**	−**0.449**	−**0.529**	−**0.520**
**Visibility**	**Wind direction**	−**6.007**	**342**	**<0.001**	−**0.402**	−**0.210**	−**0.309**	−**0.319**
Visibility	Solar radiation	1.984	279	0.048	0.001	0.232	0.118	0.113
Pluviosity	Wind speed	−0.464	462	0.643	−0.112	0.070	−0.022	−0.154
**Pluviosity**	**Wind direction**	**7.820**	**462**	**<0.001**	**0.259**	**0.420**	**0.342**	**0.353**
Wind speed	Wind direction	8.698	472	<0.001	0.291	0.447	0.372	0.187
Wind direction	Solar radiation	−5.321	397	<0.001	−0.347	−0.164	−0.258	−0.288
Tidal amplitude	Lunar day	−5.711	516	<0.001	−0.323	−0.161	−0.244	−0.229

Included are results for t-statistics, degrees of freedom (d.f.), *p*-value, upper and lower limits of 95% confidence intervals for Pearson’s product-moment correlation coefficient, *ρ,* (LL and UL, respectively), sample correlation coefficient (*r*), and Spearman’s rank correlation coefficient (*s*). Covariates exhibiting high, possible problematic correlations are typed in boldface.

#### 1. The blacknose shark, *Carcharhinus acronotus*

**Figure 8:**
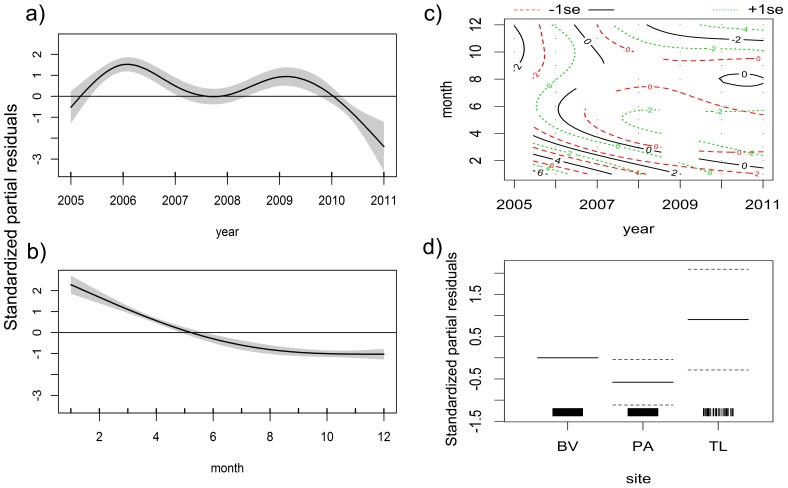
The *SPT* model for the blacknose shark. Spatiotemporal zero-inflated generalized additive models (ZIGAM) of blacknose shark abundance off Recife, comprising the *SPT1* model of the additive effects of a) year and b) month fitted with independent smooth functions, c) the *SPT2* model of the interacting effects of year and month fitted with the same smooth function, and d) the spatial effects of the three sampling sites, namely Boa Viagem (BV) and Paiva (PA), both nearshore, and the middle continental shelf (CS). The horizontal lines, the nonlinear lines and the shaded area in a) and b) depict null effects, smooth functions and 95% confidence intervals, respectively. The solid and dashed lines in c) depict isolines of standardized partial residuals and 95% confidence intervals, respectively. The solid and dashed horizontal lines in d) depict effect coefficients and 95% confidence intervals, respectively.

**Figure 9:**
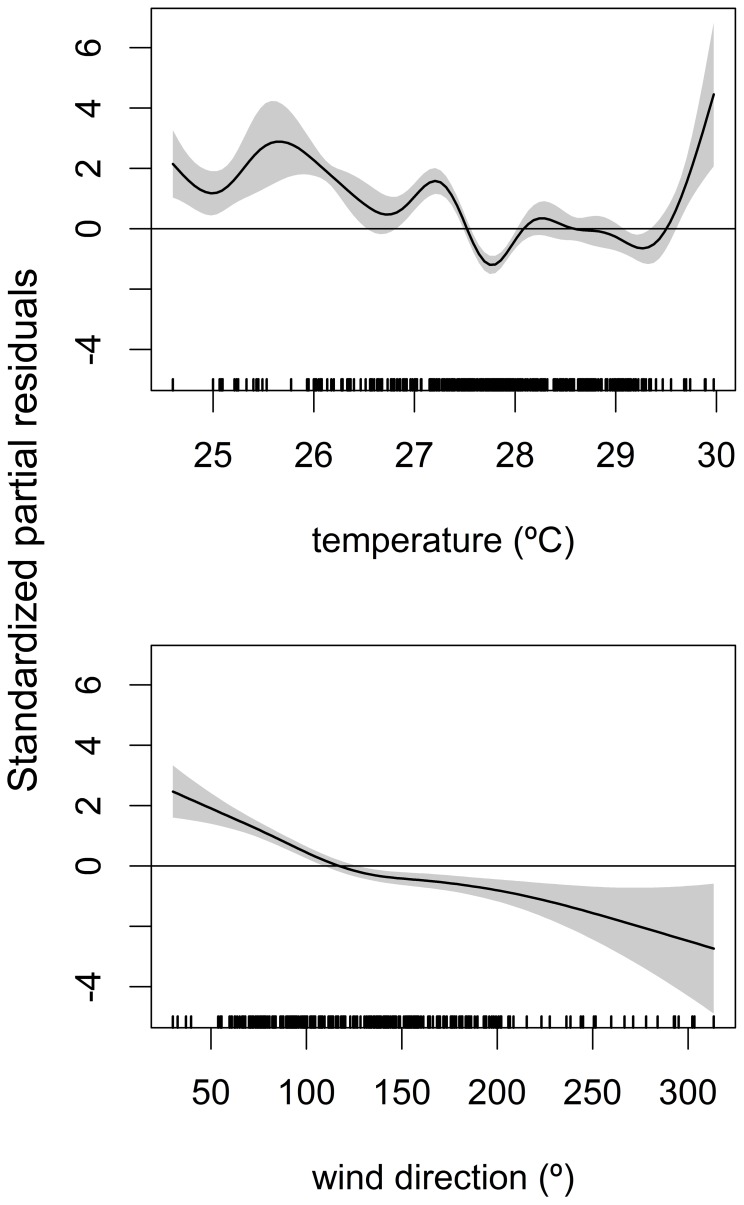
The *ENV* model for the blacknose shark. Environmental ZIGAM of blacknose shark, *Carcharhinus acronotus*, abundance off Recife, depicting the smooth functions that measure the effects of sea surface temperature (top) and wind direction (bottom) on catch rates.

The *SPT1* model revealed a decline in blacknose shark abundance from 2006 through 2011, although the year 2009 hampered an otherwise monotonous depletion (Fig. 8a). This species exhibited a clear seasonality, being more abundant during the first semester (Fig. 8b). However, the *SPT2* model showed that it became particularly absent from September through May in more recent years (Fig. 8c). Both predictors *year* and *month*, as well as the interaction between them, were important to explain the variability in blacknose shark catch (Table 5). Regarding spatial distribution, the catch rate was highest in the middle continental shelf (CS) and lowest in PA (Fig. 8d). PA showed significantly lower catch rates than BV (Z = −2.141; *p* = 0.032) but no differences were found between CS and BV (Z = −1.517; *p* = 0.129). Overall, the *SPT1* model seems to fit the data better than *SPT2* due to higher adjusted coefficient of determination, *R^2^_adj_*, and higher percentage of explained deviance (Table 5). The *ENV* model selected *temp* and *winddir* as the best predictors of blacknose shark abundance (Table 6), which was higher when temperatures were lower than 27.5°C and when wind was blowing from northern and eastern quadrants (Fig. 9).

**Table 5:** Summary of *SPT* models of shark abundance.

Species	Model	Predictor	edf	Ref.df	Chi.sq	*p*-value	*R^2^_adj_*	Dev.exp.
*C. acronotus*	*SPT1*						0.558	40.3%
		*year*	4.519	4.887	25.82	<0.001		
		*month*	1.888	1.987	44.74	<0.001		
	*SPT2*		5.874	5.994	81.84	<0.001	0.464	33.4%
*G. cirratum*	*SPT1*						0.233	13.7%
		*year*	1.748	2.113	16.11	<0.001		
		*month*	5.654	6.821	13.74	0.0512		
	*SPT2*		5.734	5.973	25.01	<0.001	0.236	12.1%
*G. cuvier*	*SPT1*						0.415	38.5%
		*year*	1.951	1.996	32.55	<0.001		
		*month*	6.320	7.607	22.54	0.0031		
	*SPT2*		10.46	10.92	61.04	<0.001	0.544	47.5%

*SPT1* models approach the additive effects of *year* and *month* with independent smooth functions, whereas *SPT2* models approach the interacting effects of *year* and *month* with the same smooth function. Included are the species names, the predictor variables, the effective degrees of freedom (edf) and reference degrees of freedom (Ref.df), the χ^2^-statistics value (Chi.sq), the *p*-value, the adjusted coefficient of determination (*R^2^_adj_*), and the percentage of null deviance explained by the model (Dev.exp.).

**Table 6:** Summary of *ENV* models of shark abundance.

Species	Model	Variable	edf	Ref.df	χ^2^-stat.	*p*-value	*R^2^_adj_*	Dev.exp.
*C. acronotus*	*temp*+*winddir*						0.478	44.6%
		*temp*	10.66	12.15	36.71	<0.001		
		*winddir*	2.682	3.426	18.89	<0.001		
*G. cirratum*	*visib*	*visib*	2.428	2.974	16.46	<0.001	0.269	13.8%
*G. cuvier*	*tidamp*+*pluvio*						0.215	28.5%
		*tidamp*	3.856	3.985	20.85	<0.001		
		*pluvio*	3.153	3.895	10.86	0.0261		

Included are the species names, the final *ENV* models assessed by forward selection, the predictor variables composing the *ENV* model, the effective degrees of freedom (edf) and reference degrees of freedom (Ref.df), the χ^2^-statistics value (χ^2^-stat.), the *p*-value, the adjusted coefficient of determination (*R^2^_adj_*), and the percentage of null deviance explained by the model (Dev.exp.).

#### 2. The nurse shark, Ginglymostoma cirratum

**Figure 10:**
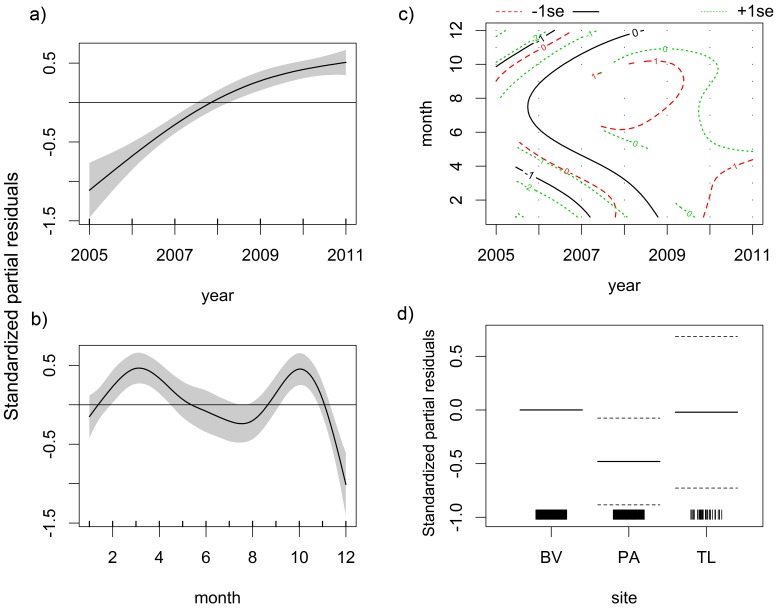
The *SPT* model for the nurse shark. Spatiotemporal ZIGAMs of nurse shark, *Ginglymostoma cirratum*, abundance off Recife, comprising the *SPT1* model of the additive effects of a) year and b) month fitted with independent smooth functions, c) the *SPT2* model of the interacting effects of year and month fitted with the same smooth function, and d) the spatial effects of the three sampling sites, namely Boa Viagem (BV) and Paiva (PA), both nearshore, and the middle continental shelf (CS).

**Figure 11:**
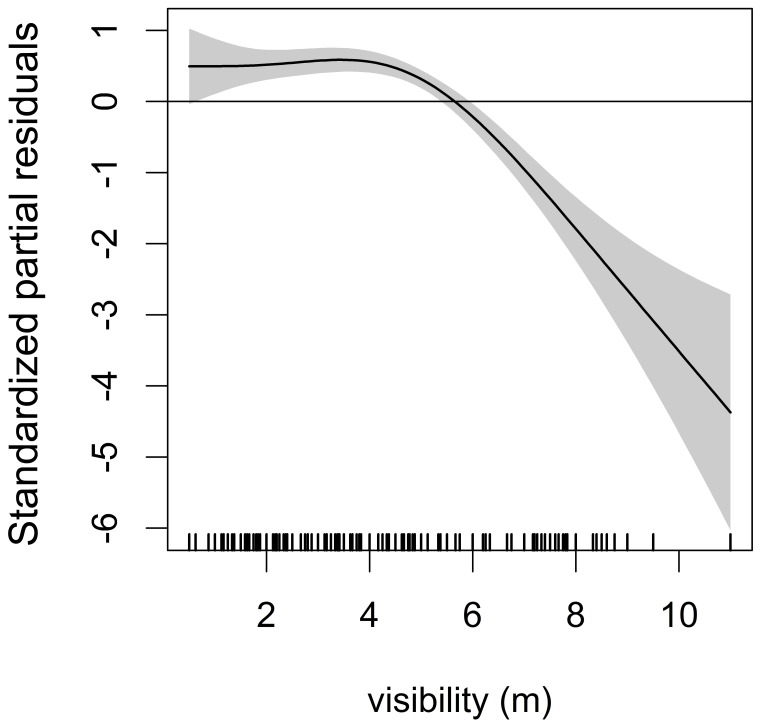
The *ENV* model for the nurse shark. Environmental ZIGAM of nurse shark, *Ginglymostoma cirratum*, abundance off Recife, depicting the smooth function that measure the effect of visibility on catch rates.

Nurse shark abundance increased monotonically from 2005 through 2011 (Fig. 10a). Seasonality in abundance was not clear, but higher abundances were found between February and April and around October (Fig. 10b). The *SPT2* model showed that nurse sharks were more abundant from June to October in the first years of surveying but they also became frequent between January and April since 2009 (Fig. 10c). The predictor *year* and the interaction between *year* and *month* produced significant effects on abundance (Table 5). Regarding spatial distribution, PA showed significantly lower numbers of nurse sharks compared to BV (Z = −2.377; *p* = 0.017) but no differences in abundance were observed between CS and BV (Z = −0.061; *p* = 0.952) (Fig. 10d). Yet, the *R^2^_adj_* values of both *SPT1* and *SPT2* models and the percentage of explained deviance were low (Table 5). The *ENV* model selected *visib* to predict nurse shark abundance, with higher abundances occurring at lower visibilities (Fig. 11), but this model also yielded a low *R^2^_adj_* value and explained a small amount of deviance (Table 6).

#### 3. The tiger shark, *Galeocerdo cuvier*

**Figure 12:**
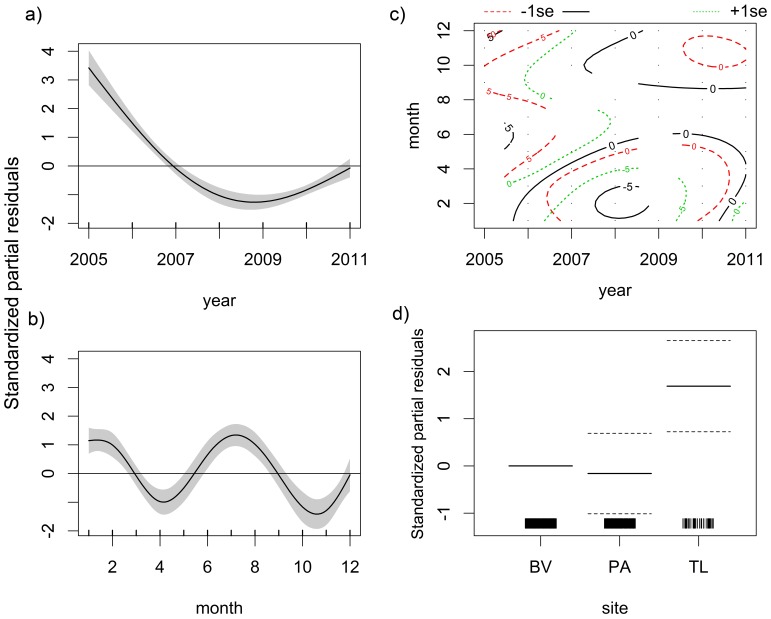
The *SPT* model for the tiger shark. Spatiotemporal ZIGAMs of tiger shark, *Galeocerdo cuvier*, abundance off Recife, comprising the *SPT1* model of the additive effects of a) year and b) month fitted with independent smooth functions, c) the *SPT2* model of the interacting effects of year and month fitted with the same smooth function, and d) the spatial effects of the three sampling sites, namely Boa Viagem (BV) and Paiva (PA), both nearshore, and the middle continental shelf (CS).

**Figure 13:**
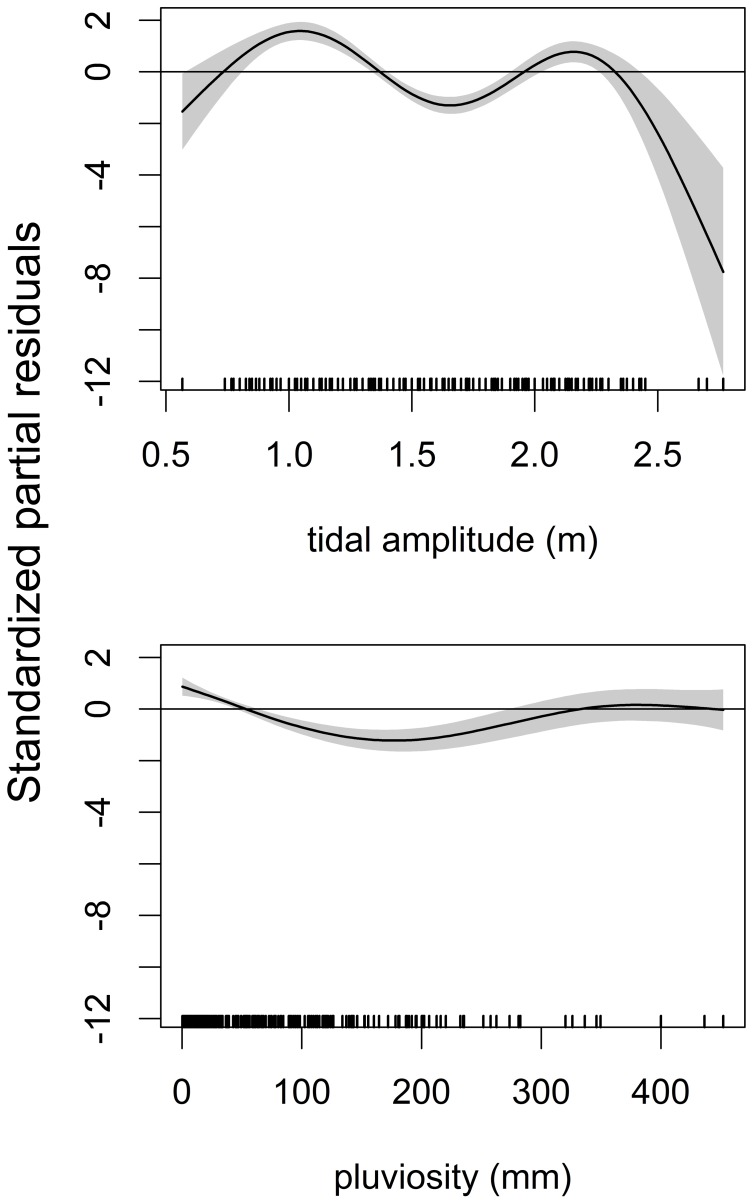
The *ENV* model for the tiger shark. Environmental ZIGAM of tiger shark, *Galeocerdo cuvier*, abundance off Recife, depicting the smooth functions that measure the effects of tidal amplitude (top) and pluviosity (bottom) on catch rates.

Tiger shark abundance declined considerably from 2005 to 2009, but it increased from 2009 onwards (Fig. 12a). Higher abundances spanned from January to March and from June to September (Fig. 12b). However, the *SPT2* model revealed that seasonal peaks of abundance occurred from April to August and from October to December during the first three years of surveying, but in subsequent years an absence of tiger sharks was observed, particularly between September and May (Fig. 12c). This absence was temporally precise and is depicted as a roughly elliptical array of negative isolines centered at about February 2008 and spanning from 2006 through 2010, although low abundances were still observed in the last quarter of 2011. Both *year* and *month* and the interaction between them produced significant effects on abundance (Table 5). Regarding spatial distribution, tiger sharks were most abundant in CS and least abundant in PA (Fig. 12d), with significant differences being found between CS and BV (Z = 3.499; *p*<0.001), but not between PA and BV (Z = −0.378; *p* = 0.706). Confidence intervals of CS and PA do not superpose hence there is also evidence that CS and PA effects are different. The *SPT2* model had higher *R^2^_adj_* and percentage of explained deviance than *SPT1* (Table 5). The *ENV* model selected both *tidamp* and *pluvio*, with higher tiger shark abundances being associated with low or high tidal amplitudes and low pluviosity (Fig. 13), although the resulting *R^2^_adj_* value and percentage of explained deviance were low (Table 6).

## Discussion

Understanding the composition and dynamics of shark populations in nearshore waters is essential to promote their conservation and predicting environmental responses to human pressure. The ecological significance of elasmobranchs warrants the sustainable management of their populations, which can only be achieved with adequate information on their ecology. Yet, the elasmobranch communities from the western South Atlantic remain poorly known. The species surveyed in this region include a considerable diversity of sharks and batoids [42] which are known to occur in tropical regions [59]–[62]. The shark assemblage was clearly dominated by two coastal species (i.e. the blacknose and nurse sharks) and the tiger shark, which uses both coastal and oceanic habitats. Tiger sharks are circumglobal at tropical latitudes and nurse sharks occur in the tropical Atlantic and eastern Pacific Oceans, whereas blacknose sharks occur exclusively in the tropical western Atlantic Ocean [61], [63]. The distribution of these species also differs in nurse sharks being sluggish bottom-dwellers, thus spending most time in association with the benthos [60]–[61], and blacknose sharks relying on RAM ventilation to breathe [64] and, similarly to tiger sharks, being associated mainly to the water column. The remaining species were rare except for the bull, *Carcharhinus leucas*, and the blacktip, *C. limbatus*, sharks which were caught more frequently but still in low numbers.

The blacknose sharks sampled were mostly adult and subadult individuals because this species matures at ∼100 cm TL in this region [65]. Hook-selectivity could have precluded the adequate survey of younger stages since artisanal fishermen catch small juveniles with gillnets in nearby regions [66]. In the North Atlantic, blacknose sharks use nearshore waters during their whole life-cycle [10], [67]–[68] but smaller juveniles seem to use waters <10 m in depth [61], [68], which corresponds to the area where drumlines operated off Recife. Despite both sexes being equally represented in the catch composition, the quarterly variation in the sex ratio suggests that females may leave the study area during the first quarter, i.e. mid to late austral summer. Male-biased blacknose shark catches were also reported during the first semester off North Carolina [68], although this period corresponds to winter and spring boreal seasons. Nurse shark size-structure off Recife was wide-ranging but young juveniles were not caught, suggesting either hook-selectivity or spatial segregation of younger juveniles. Nurse sharks measuring 50−120 cm TL were reported to inhabit shallow coral reefs and grass flats [69], which do not exist off Recife. Nurse shark size at first maturity is about 214 and 227 cm TL for males and females, respectively [69], thus most sharks were juvenile and most mature sharks were female. This distribution seems to agree with the trend observed in another region off northeastern Brazil [70]. Furthermore, the quarterly variation in sex ratio suggests that males tend to leave the study area particularly in the third quarter, which has been confirmed with acoustic telemetry [71]. As for tiger sharks, juveniles comprised the bulk of the catch because only two individuals were as large as the reported size-at-maturity of 310−320 cm TL [72]. Compared to smaller juveniles, large tiger sharks could have more chance of biting off through the hook or leader and escaping the longline, yet the gear used in this study is believed to have minimized such occurrences. Indeed, such gear bias would expectedly result in a gradual decline in the catch rate of larger individuals, whereas the catch rate of sharks >200 cm TL dropped suddenly and kept invariably low through sizes >350 cm TL. Tiger sharks >200 cm TL should thus use this habitat less frequently or be less prone to taking the baited hook, and the former seems more likely. Nonetheless, the coinciding occurrence of individuals measuring about the reported size at birth of 70−90 cm TL [72]–[73] and mature sharks exclusively during the first quarter suggests that neonates could be born during this period. The subsequent modal progression in size-frequencies between the first and the third quarters should reflect growth because tiger sharks seem to grow at compatible rates in this region [74].

The spatiotemporal modeling of species abundance showed some interesting trends. Blacknose shark abundance declined considerably between 2006 and 2011, whereas nurse shark abundance increased since 2005. The blacknose shark has been previously reported as one of the most abundant shark species off Recife, with catch rates equaling 0.29 individuals per 100 hooks [75], and it was the most abundant species during the first years of sampling when nurse sharks were less abundant [42]. However, this pattern reversed as nurse shark catch rates increased monotonically up to one order of magnitude along the years and blacknose sharks became infrequent in the catch composition [42]. In this survey, blacknose sharks experienced high (∼80%) relative mortality and nurse sharks had virtually zero mortality [42]. Yet, since only 120 blacknose sharks have been removed by this 8-year survey, the observed depletion should not be ascribed to this source of mortality. Indeed, this species seems to experience high fishing pressure in some areas of its range [76] and considerable declines in abundance have been reported for the northern hemisphere mostly since 2000, with recent assessments estimating the US population in 2006 to be at 25% of virgin levels [77]. In the south hemisphere no evidence of population decline has yet been found [78], but this region is extremely underrepresented in the fisheries literature and it seems possible that the abundance decrease off Recife could also derive from regional sources of fishing mortality.

In contrast, increasing nurse shark abundance and size range suggest that the local population of this species could be expanding. The capture of nurse sharks in Brazilian waters has been prohibited since 2004 (Brazilian Ministry of the Environment, Annex I of Normative Instruction #5, 21 May 2004), which expectedly contributes to the growth of their populations. Off Recife, such effect could have been locally exacerbated due to the continual removal of blacknose sharks by this survey since 2004, which may have increased the amount of empty habitat available to the nurse shark. The blacknose and nurse sharks are both coastal and have partially-overlapping diets [29] thus they should be ecologically-linked to some degree. Also, blacknose shark seasonality off Recife seems to partially coincide with peaks of nurse shark abundance, despite the latter occurring in this region throughout the year. Tag-and-recapture and acoustic telemetry data showed that nurse sharks are site-fidelic and resident in this region [71], evidencing the suitability of nearshore waters off Recife for nurse sharks thriving. Furthermore, both nurse and blacknose sharks seem to be less abundant in PA than in BV. This could relate to a higher habitat complexity in BV due to the presence of a shallow reef, and to the location of the Jaboatão estuary in PA’s northernmost section which expectedly deflects its plume towards BV due to the prevailing northward coastal currents. Both factors could contribute to BV being a more attractive foraging ground than PA.

Regarding tiger sharks, abundance was particularly low during a 4-year period but there is no evidence that it could be decreasing long-term. Previous studies report fluctuating annual catch rates for tiger sharks [79]–[81], with peaks of abundance occurring in periods of several years [82]. Tiger shark catch rates in the North Atlantic seem to be stable [83] or even increasing [84], contrasting with declining catch rates off Australia [81]. A longer time series is required to understand trends in tiger shark abundance in the South Atlantic. Yet, abundance seasonality was detected as it drops considerably from October onwards. Although the *SPT2* model performed better than *SPT1* for this species, thus suggesting a possible shift in seasonality, such trend was mostly shaped by the seemingly temporary absence of tiger sharks during periods in which they were abundant during the first few years of surveying. Additional sampling is thus required to clarify abundance seasonality in tiger sharks off Recife. Tiger sharks off western Australia seem to be most abundant from June to August [85], whereas they reside year round off Florida and seasonally migrate north as far as Nova Scotia [86]. Given that early-juvenile tiger sharks have high growth rates [74], the abundance pattern off Recife suggests that young-of-the-year use neritic habitats for ∼9 months to enhance growth and further move to other regions or depths after attaining a size of 150−200 cm TL. Tiger shark catch rates in the western North Atlantic have been positively correlated with depth [84], and in this study they were more abundant in waters from the middle continental shelf than in nearshore waters. Interestingly, and in opposition with the two coastal species (i.e. the blacknose and nurse sharks), tiger sharks did not seem to prefer any of the nearshore sampling sites. This species could thus be using deeper waters as they grow larger, although they will probably still move regularly to shallow, inshore waters to forage [7]. On the other hand, satellite tagging has shown that tiger sharks in this region use both the neritic and oceanic provinces [41], [87]–[88], thus these juveniles could also be moving to oceanic waters after attaining an adequate size, as suggested by low numbers of sharks ≥200 cm TL.

The environmental modeling selected sea surface temperature, tidal amplitude, wind direction, visibility, and pluviosity for predicting species abundance. Temperature and tidal amplitude have been reported to influence the distribution and abundance of sharks in coastal habitats [16], [89]–[90]. Pluviosity may influence shark abundance in coastal areas close to estuaries because it increases freshwater runoff and estuarine drainage, which could also have an effect on visibility. The wind direction shapes a number of environmental features off Recife, including the direction of coastal currents, pluviosity and water visibility. Overall, the estimated spatiotemporal and environmental models showed a reasonable fit for blacknose and tiger sharks. Despite a low fit, the amount of deviance explained (13−14%) for nurse sharks was nevertheless higher than those from other studies (e.g., [84]). By comparing the performance of ZIGAM and COZIGAM, it was possible to test if the regular component of the model depended on the probability of non-zero-inflation, which would reflect the mechanistic nature of the zero-inflation process and promote estimation efficiency by reducing the number of parameters in the model (Liu & Chan 2010). The fact that ZIGAM outperformed COZIGAM indicates that the zero-inflated and the regular processes were generally independent. However, other approaches could perhaps perform better for the nurse shark, such as the partially-constrained ZIGAM that assumes proportionality constraints to some, not all, covariates [91].

The conservation of elasmobranch communities in nearshore waters is of utmost importance for the long-term sustainability of coastal ecosystems. However, understanding the bioecological processes that regulate shark abundance and distribution is required to ensure adequate management of shark populations. In this study, interspecific variability in abundance dynamics across spatiotemporal and environmental gradients suggest that the ecological processes regulating shark abundance off Recife are relatively independent between species. If so, this could add a considerable amount of complexity to fisheries management under a multi-species framework, leading to the need of extending the current knowledge on shark ecology. This study contributed to our understanding of the species-specific dynamics of three coastal sharks in a region virtually unknown to fisheries and marine sciences. However, further research conducted at wider geographical regions in the South Atlantic is required in order to understand the relationship between the trends observed in the studied area and those exhibited by the whole populations of these species.
